# Cell density overrides the effect of substrate stiffness on human mesenchymal stem cells’ morphology and proliferation†
†Electronic supplementary information (ESI) available. See DOI: 10.1039/c7bm00853h


**DOI:** 10.1039/c7bm00853h

**Published:** 2018-03-12

**Authors:** Balu Venugopal, Pankaj Mogha, Jyotsna Dhawan, Abhijit Majumder

**Affiliations:** a Institute of Stem Cell Biology and Regenerative Medicine , Bangalore 560065 , India . Email: abhijitm@iitb.ac.in; b Dept. of Chemical Engineering , IIT Bombay , Mumbai , 400076 India; c CSIR-Centre for Cellular and Molecular Biology , Hyderabad , 500007 India

## Abstract

The effect of substrate stiffness on the cellular morphology, proliferation, and differentiation of human mesenchymal stem cells (hMSCs) has been extensively researched and well established. However, the majority of these studies are done with a low seeding density where cell to cell interactions do not play a significant role. While these conditions permit an analysis of cell–substratum interactions at the single cell level, such a model system fails to capture a critical aspect of the cellular micro-environment *in vivo*, *i.e.* the cell–cell interaction *via* matrix deformation (*i.e.*, strain). To address this question, we seeded hMSCs on soft poly-acrylamide (PAA) gels, at a seeding density that permits cells to be mechanically interacting *via* the underlying substrate. We found that as the intercellular distance decreases with the increasing seeding density, cellular sensitivity towards the substrate rigidity becomes significantly diminished. With the increasing seeding density, the cell spread area increased on a soft substrate (500 Pa) but reduced on an even slightly stiffer substrate (2 kPa) as well as on glass making them indistinguishable at a high seeding density. Not only in terms of cell spread area but also at a high seeding density, cells formed mature focal adhesions and prominent stress fibres on a soft substrate similar to that of the cells being cultured on a stiff substrate. The decreased intercellular distance also influenced the proliferation rate of the cells: higher seeding density on the soft substrate showed cell cycle progression similar to that of the cells on glass substrates. In summary, this paper demonstrates how the effect of substrate rigidity on the cell morphology and fate is a function of inter-cellular distance when seeded on a soft substrate. Our AFM data suggest that such changes happen due to local strain stiffening of the soft PAA gel, an effect that has been rarely reported in the literature so far.

## Abstract 2

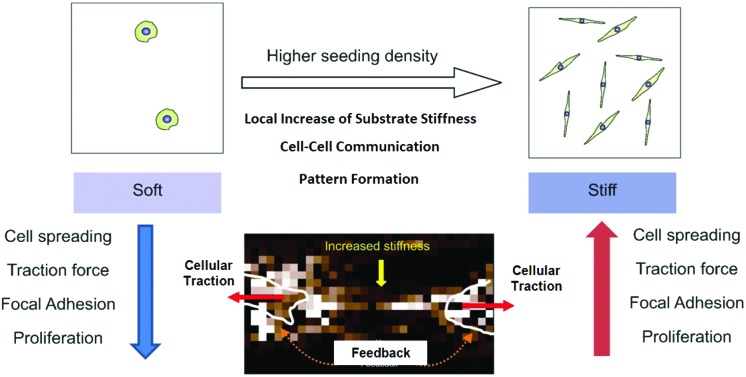
Cell–cell interaction *via* substrate deformation in turn modifies cellular response to substrate rigidity.

## Introduction

Mechanical signals from the micro-environment are crucial during development and for the maintenance of healthy tissues.^1^ Malfunctioning in mechano-signalling processes has been shown to contribute to many pathological conditions.^2^,^3^ Out of many possible mechano-signals, the most well studied one is substrate/tissue stiffness. *In vitro* studies have shown that on a soft substrate, adherent cells spread less, remain soft and less contractile, do not produce mature focal adhesions and actin stress fibres and show an altered nuclear morphology.^4^–^6^ Studies with mesenchymal stem cells (MSCs) have shown that the differentiation process can be controlled using substrates of tissue-specific stiffness.^7^ It has also been demonstrated when human MSCs (hMSCs) are cultured on a very soft substrate, they exit the cell cycle and go into a reversibly arrested state known as quiescence.^8^

Typically, most of these *in vitro* studies employ sparse cell seeding to permit the analysis of activities such as the dynamics of cell–substratum interactions at the single cell level. However, cells in a tissue are neither completely isolated nor in a uniform monolayer (except for epithelia), but often groups of cells remain connected through the ECM. We also know that adherent cells create a strain field around themselves by exerting acto-myosin contractile stresses.^9^,^10^ Thus, cells in a tissue may possibly interact with each other *via* the strain field created by an individual cell causing deformation of the underlying matrix.^11^,^12^ In such situations, the extent of that field is likely to depend on both cellular and substrate properties, which in turn may influence the behaviour of neighbouring cells in the group. Understanding this aspect of force-mediated intercellular communication is fundamental in building models of tissue development, homeostasis, and morphogenesis.

In one of the first studies in this area, Reinhart-King *et al.* in 2008 had shown that when endothelial cells (ECs) are seeded on a soft substrate, they create a strain field that is strong enough to hinder the movement of neighbouring cells, and limits the separation distance between the interacting cells.^13^ ECs, seeded above a critical density, were shown to form ring-like networks depending on substrate stiffness and ligand density.^14^,^15^ In a similar study, it was observed that two cells at a sufficiently large distance (>300 μm) can sense each other, elongate and finally form the connection.^16^ This observation suggests that such force signalling *via* the matrix may work in a quite long range. However, how such communication takes place is still debated and unresolved.^17^,^18^ Another gap in the field is that all of these reports considered cellular morphology as the only read out, and the effect of cell–cell force interaction *via* the deformable matrix on the cellular fate or function is yet to be explored.

In this study, we explored the behaviour of hMSCs when plated on a soft substrate at a high yet sub-confluent seeding density. We show that depending on the distance between the neighbouring cells, the mechano-response of a cell may vary dramatically. We found that (a) the cell–cell distance has profound effects on cellular responses to mechano-signals, (b) at a sufficiently high seeding density, even on a soft substrate cells spread, form stress fibres and mature focal adhesions, (c) such cell–cell mechanical interactions can switch the cellular state from one to other such as from quiescence to proliferation, and (d) effective stiffness of the substrate between two cells increases due to the applied strain. Our findings indicate that closely spaced cells sense the tension caused by their neighbours, and start spreading in response. In summary, cell crowding and the resultant increase in apparent stiffness due to cellular traction can override many of the known effects of substrate stiffness on the cellular morphology and functions.

## Experimental section

### Substrate preparation

Polyacrylamide gels (PAA) of different stiffness were prepared by cross-linking 40% poly-acrylamide and 2% bis-acrylamide solution, as described.^19^ Protocols for substrate preparation and modulus values were adopted from a published work.^20^ Briefly, the gels were prepared between two glass coverslips, one coated with 3-APTMS (Sigma) and the other with octadecyl-trichlorosilane (Sigma-Aldrich). After gelation, the non-adherent plate was removed. The gel was coated with type I collagen (50 μg ml^–1^) (Advanced Biomatrix) using sulfo-SANPAH based conjugation at 4 °C overnight.^5^ The control glass coverslips were also coated with Type 1 collagen. Throughout our experiments, the gel thickness was controlled by controlling the volume of gel solution placed between the coverslips. Before cell seeding, any excess collagen was washed off and the gel was equilibrated with media for one hour.

### Cell culture

Primary hMSCs, obtained from Texas A&M University at passage 1, were used for all experiments with the prior approval of the InStem Institutional Committee on Stem Cell Research, IIT Bombay institutional ethical committee and in accordance with the ICMR-DBT stem cell research guidelines. All cell culture reagents were obtained from Gibco™ Invitrogen unless otherwise mentioned. All experiments were carried out with early passage hMSCs (passage 2–passage 5). hMSCs were expanded in alpha MEM containing 16% MSC certified FBS, 1% glutamax and 1% penicillin–streptomycin, maintained at 37 °C with 5% CO_2_. hMSCs were seeded onto the 2D polyacrylamide gel substrate with a pre-defined seeding density in 100 μl media and flooded after 2 hours.

### Cell density and average cell to cell distance estimation

After 1 h of cell seeding on the substrate of choice, unattached cells were washed off with excess media. Then, after additional 3 h, the nuclei were stained with Hoechst (1 : 10 000) and imaged. For quantification, at least 16 images were taken for each gel. These images were used to estimate the average seeding density. To obtain the average distance between two neighbouring cells, the average area available per cell was considered as a circle. The distance between the two neighbouring cells was then calculated as the diameter of this circular area.

### Calcein AM staining

Calcein AM (Cat no C3099. Molecular Probes® Invitrogen) was used to determine the cell viability and cell morphology. Cells were stained with Calcein AM and Hoechst in freshly prepared serum free Opti-MEM (11058021) containing Calcein AM (dilution 1 : 2000) and Hoechst (dilution 1 : 10 000). The cells were then incubated for 15 minutes at 37 °C and imaged using an Axiovert 40 CFL (Carl Zeiss) or an EVOS® FL Auto cell imaging system (Life Technologies).

### BrdU staining

The BrdU incorporation assay was used to identify proliferating cells. Cells cultured on gels and TCP after 48 h of seeding were incubated with medium containing a BrdU labeling agent in a dilution 1 : 1000 (Roche Diagnostics) for 2 h. Cells were then fixed with 4% paraformaldehyde, permeabilized with 0.5% Triton-X-100 (Sigma Aldrich) for 5 minutes and blocked with 10% horse serum (Gibco® Invitrogen). The anti-BrdU antibody (DSHB, University of Iowa) was used to target the BrdU labelling agent at a dilution 1 : 50. Fixed cells were incubated for 1 h, the unbound primary antibody was washed off and the bound antibody was detected using an Alexa Fluor® 568 goat anti-mouse antibody. Labeled cells were imaged using an Axio Scope A1 (Carl Zeiss). DAPI was used to stain the nucleus.

### Actin and vinculin staining

hMSCs were fixed with an ice cold mixture of 1 : 1 (v/v) (4% paraformaldehyde (PFA)) pH 7: permeabilizing buffer (1% Triton-X-100-Sigma Aldrich) for 1 minute on ice. Cells were then washed twice with cytoskeleton stabilizing buffer (CSB) (60 mM PIPES, 27 mM HEPES, 10 mM EGTA, 4 mM magnesium sulphate (heptahydrate), pH 7) and fixed again with only 4% PFA for 5 min on ice. Cells were again washed thrice with CSB and blocked with 1.5% BSA supplemented with 0.5% Triton X-100 for 30 min on ice. The anti-vinculin antibody (Cat. No. V9131, mouse monoclonal, Sigma) was used at a dilution 1 : 400 (diluted in blocking buffer) and incubated overnight at 4 °C. Vinculin was then detected with an Alexa Fluor® 568 rabbit anti-mouse antibody (Cat. No. A11061, Thermo scientific) at a dilution of 1 : 1000. Cells were then simultaneously incubated with an Alexa Fluor® 488 Phalloidin (Cat. No. A12379, Thermo scientific) as 1 : 400 dilution and with Hoechst 33342 (Cat. No. H3570) with a dilution of 1 : 10000. The cells were imaged using a laser scanning confocal microscope (LSM, Carl Zeiss, objective 10×).

### Live cell imaging

Changes in the cellular morphology and network pattern upon addition of LatB were captured in real time. The cell seeded gels were fixed on the microscope stage and latrunculin B (0.5 μM) was added through the side wall without disturbing the gels. The effect of LatB was recorded manually using an Axiovert 40 CSL microscope.

### Atomic force microscopy (AFM)

Atomic force measurements were done with a TR800PB silicon nitride pyramidal tip probe on MFP-3D (Asylum Research) under contact mode force. The spring constant of a cantilever ranged from 0.09 to 0.27 N m^–1^ and a frequency from 17 to 28 kHz. Force maps were taken for a maximum of 90 × 90 μm^2^ with at least 20 lines, each having at least 20 readings and a maximum of 32 lines and 32 readings on each line. The force maps were then fitted with the hertz model provided within the software from Asylum research after setting the correct parameters for tip geometry, material of the probe and the Poisson's ratio of the material. For the LatB study under AFM, force-maps were taken for the selected region before and after adding the LatB.

### TFM

Gels of 500 Pa were made on 22 × 22 mm^2^ coverslips, once the gels were solidified, 25 μl of 500 Pa solution having 1 μm fluorescent beads (Fluka with a final concentration of 1 : 50) along with APS and TEMED was added on the hydrophobic plate, then the solidified gel was inverted onto the top of it and allowed to solidify. The gels were then treated with sulfo-SANPAH and coated with Collagen type-I as mentioned above. After 24 h of cell seeding, the cells were lysed using Triton-X 100 without disturbing the gels, images of cells in the phase were taken before adding Triton-X, and the images of fluorescent beads were taken before and after adding Triton-X, using the EVOS FL Auto cell imaging system (Invitrogen). The code from J. P. Butler^21^ was used to calculate the bead displacement and traction force.

## Results and discussion

### Combinatorial effect of substrate stiffness and cell seeding density on cellular morphology

To explore the combinatorial effect of substrate stiffness and inter-cellular distance, we cultured hMSCs at different seeding densities (1000, 2000, 4000, 8000, and 16 000 cells per cm^2^) on collagen coated soft PAA gels of different stiffness (Young's moduli 500 Pa, 1 kPa, 2 kPa) and glass. Although we did not have a precise control over distance between every pair of cells, with increasing seeding density, the average inter-cellular distance decreases (Fig. S1†). At a low seeding density, such as 1 K cm^–2^ the average distance between the two cells is approximately 300 μm which reduces to 150 μm or less at a seeding density of 4 K or higher. The average distance was estimated from microscopy images as described in the Experimental section. Fig. S1C† confirms that the average number of cells adhering to the substrate, and thus the average distance between two neighbouring cells, does not depend on substrate stiffness.

**Fig. 1:**
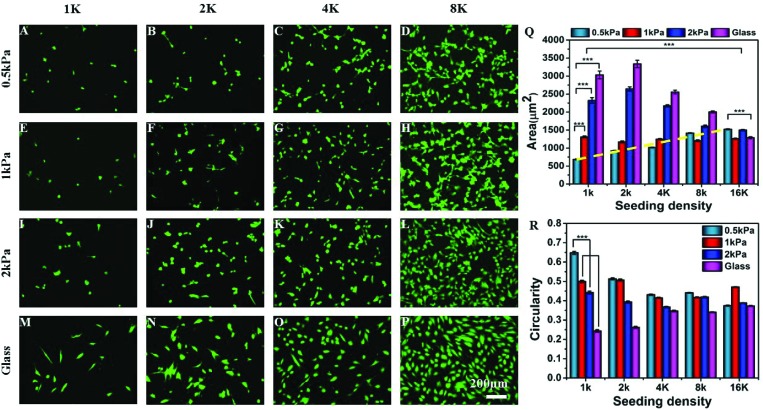
Combinatorial effect of seeding density and substrate stiffness: (A–P) cells, stained with calcein AM, are shown on substrates of different rigidities (along the columns) and at different seeding densities (along the rows). (A) On a soft gel, sparsely seeded cells (1000 cells per cm^2^) do not spread. However, with increasing seeding density for a soft substrate (A–D: 1000–8000 cells per cm^2^) or with increasing substrate stiffness (A, E, I, and M) for a low seeding density, cell spreading increases. Other combinations show a mixed effect as quantified in (Q). (Q and R) The bar charts show the combinatorial effect of seeding density and substrate stiffness on the cell spread area and cell shape, respectively, circularity = 1 indicates completely circular cell and circularity = 0 indicates a straight line. Reported as mean ± standard error, at least 500 cells were considered from 5 independent experiments, (*P* < 0.001).

We observed that the effect of substrate stiffness on cell spreading becomes drastically modified depending on the seeding density (or inter-cellular distance) (Fig. 1). For a low seeding density, the cell area increased with substrate rigidity as shown by many researchers previously.^6^ However, this observation changed significantly as we increased the cell density. For example, at a low seeding density and a low substrate modulus, cells did not spread and took a round morphology (Fig. 1A). As we increased the seeding density, cell spreading on a very soft gel (500 Pa) increased as shown in the fluorescent microscopy images (Fig. 1A–D) and the dashed line in Fig. 1Q. We observed that the average cell spreading increased by 3 times from 500 μm^2^ to almost 1500 μm^2^ when the seeding density was changed from 1000 cells to 8000 cells per cm^2^ on a 500 Pa gel. After that, increasing the seeding density to 16 K had a minimal effect on cell spreading. By contrast, on stiffer substrates, such as 2 kPa gel (which is still within the domain of soft) or glass, the average cell spread area decreased with the increasing seeding density due to cell crowding (Fig. 1I–Q). For an intermediate stiffness of 1 kPa, these two effects counterbalance each other and an almost unaltered cell spreading at different densities was observed. Likewise, substrate stiffness and seeding density had opposing effects on the cell shape as well, as captured using cellular circularity as the measure in Fig. 1R. Circularity is defined as (4π × area/perimeter^2^) and signifies the extent of cell polarization (a fully circular projected area has circularity as 1, while a linear geometry has a circularity of 0). We observed that while cells at a low density on a soft substrate assumed a round morphology with high circularity, increasing either stiffness or density made cells more polarized. These observed combined effects of substrate stiffness and cell density on the cellular morphology were not specific to MSCs: similar morphological changes were seen with NIH 3T3 fibroblast and C2C12 myoblast cell lines (Fig. S2†).

The possibility that this change in cellular morphology was due to the increased global concentration of secreted cytokines was ruled out by increasing the media volume keeping the cell number and density constant or by increasing the total number of cells keeping the media volume and cell density constant. Such changes are expected to modify the cytokine concentration significantly. However, neither over dilution nor increased concentration brought any major change in our observation (Fig. S3†). Although these experiments rule out the effect of the global concentration of cytokines, the possibility of a local increase of cytokine concentration cannot be eliminated.

### Directional cell spreading results into global pattern formation

**Fig. 2:**
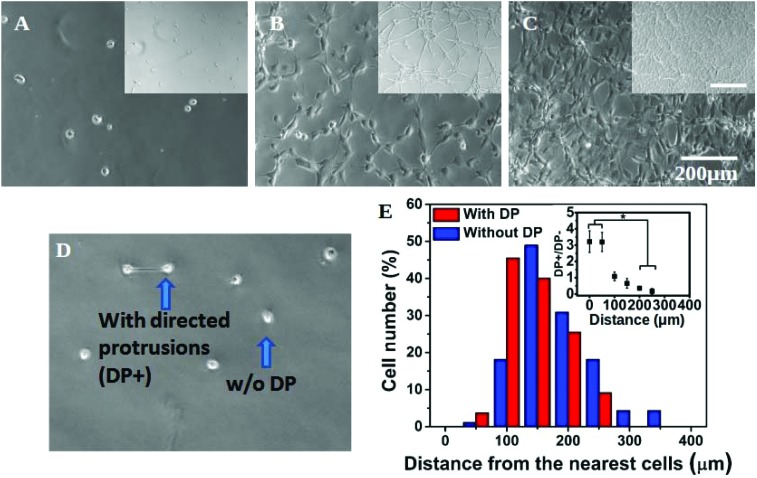
Cell–cell communication and network formation: (A–C) hMSCs on a soft gel at the seeding density 1 K, 4 K and 8 K cm^–2^ respectively. At a higher seeding density, cells start to form cell–cell contacts and create a network (B and B inset). At an even higher density, they form a monolayer (C and C inset) (inset scale 500 μm). (D) Cells at 1 K seeding density. Even at this low seeding density, a few pairs of cells form connections or extended directed protrusions (DP) towards each other. Arrows show a pair of cells with directed protrusions (DP+) and a single cell without any protrusion (DP–). (E) Cell number distribution against the distance from the nearest neighbour.

In addition to a marked effect on single cell morphology, the interplay of substrate stiffness and seeding density also gave rise to global patterning (Fig. 2A–C). In our experiments, cells were seeded as isolated and non-clustered bodies with a round morphology. However, within four hours of plating at a high density on a soft substrate, cells started to spread preferentially towards their “non-touching” neighbours (Fig. S4†) resulting into the formation of strings and networks in the next 24 hours (Fig. 2B insets and S4†). With a further increase in cell seeding density, cells completely occupied the substrate, forming a near-confluent monolayer (Fig. 2C inset and S4†). The formation of the network pattern can be dynamic, but for simplicity we did not consider time as a parameter in our study and all the observations were done 24 hours after cell seeding.

To confirm that the observed cell spreading and resulting network formation on the soft gel was indeed governed by the inter-cellular distance, we made a detailed analysis of preferential cell spreading at 1000 cells per cm^2^ seeding density on the 500 Pa gel. At this low seeding density, where the placement of cells may be considered random, we found that some cells formed directed protrusions towards their nearest neighbour (denoted as DP+) while most of the cells remained spherical (no directed protrusion, DP–). DP+ and DP– are depicted in Fig. 2D. Next, the distance of a cell from its nearest neighbour was measured and grouped under either DP+ or DP– category accordingly. The population distribution against this measured distance for each category was plotted in Fig. 2E. We found that DP– cells showed an almost normal distribution with a slight bias towards a larger separation distance (red bars). However, DP+ cells showed a strong bias towards a smaller separation distance. In other words, when cells were closely spaced (<100 μm), there was a three times higher probability for them to polarize and spread towards their nearest neighbour instead of remaining spherical (Fig. 2E and the inset). When the cell–cell distance was large, cells were more likely to remain round. We could not find any DP+ cell for a distance >300 μm. This analysis matches with our overall observation that the formation of a cellular network happens only at 4 K seeding density or higher when the intercellular distance comes down to 150 μm or lower, as shown in Fig. S1A.† This observation remained unchanged over a time period of 24 hours (Fig. S5†).

Any possibility of formation of a network in response to the uneven surface of the substrate was excluded by carefully studying surface topology by AFM (Fig. S6†).

### Cell crowding increases traction and substrate strain

**Fig. 3:**
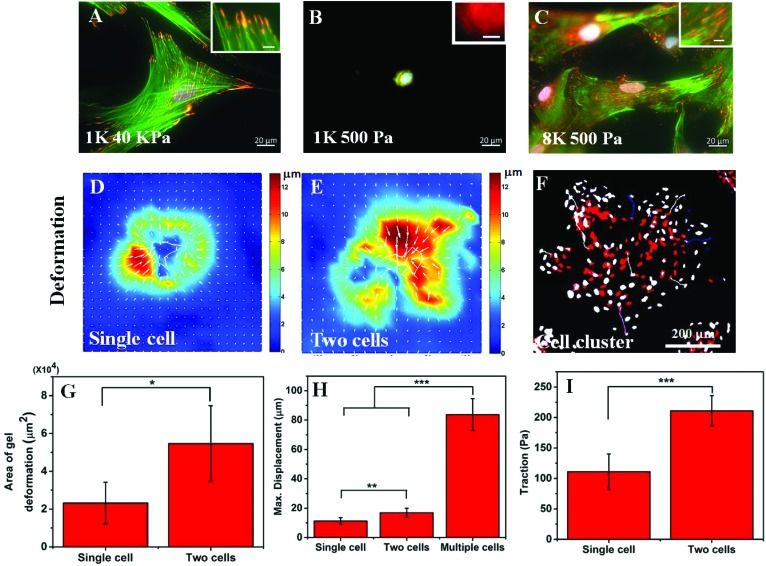
Effect of cell crowding on cellular traction: (A) hMSCs seeded on a rigid substrate (PAA gel, 40 kPA) at a low seeding density (1000 cells per cm^2^), form stress fibres (actin: green) and mature focal adhesion (vinculin: red), two hallmarks of cellular contractility. (B) When seeded on a soft substrate (PAA gel, 500 Pa), cells do not form clear stress fibres and mature focal adhesions. The absence of matured focal adhesion is evident from the diffused staining of vinculin. (C) However, when seeded at a higher density (8000 cells per cm^2^) on the soft gel, the phenotype seen on the rigid substrate is restored. Scale bar: 20 μm, inset scale bar: 5 μm. (D–E) Typical TFM heatmaps of substrate deformation for a single and a pair of cells respectively (substrate: 500 Pa PAA gel). (F) A patch of cells on the 500 Pa gel was observed in which latrunculin B was added to prevent actin polymerization and thus to decrease contractility. Here, red (pseudo-colour) and grey indicate the initial and final positions of the nuclei respectively. The coloured lines represent the displacement track of the individual nucleus from the initial to the final position. (G) The bar chart compares the total area of the substrate that experienced point deformation ≥2 μm due to the traction exerted by a single cell and two cells (500 Pa PAA gel, mean ± standard deviation., *n* = 6, *p* < 0.05). (H) Maximum point displacement of the substrate caused by a single cell, a pair of cells, and a cell cluster (500 Pa PAA gel, mean ± standard deviation, *n* ≥ 6, *p*; **<0.01 and ***<0.001). (I) Traction stress caused by a single cell, and a pair of cells (500 Pa PAA gel, mean ± standard deviation, *n* = 6, *p* < 0.001).

In the previous section, we have observed that cell spreading increases on the soft substrate when seeded at a high density. As cell spreading, traction and substrate rigidity are very closely interlinked, we also wanted to check what happens to the cellular traction with a higher seeding density. It is known that cell spreading and cellular traction both increase simultaneously with increasing substrate rigidity.^7^,^22^ However, the effect of contractility on cell spreading is strikingly opposite depending on whether cells are cultured on soft or rigid substrates. Reduction of traction using a myosin inhibitor has been shown to cause a reduction in cell spreading for rigid substrates but an increase for soft substrates.^23^ Thus, we wanted to ask whether the increase in cell spreading is a result of reduction in traction or increasing seeding modified the cellular response to substrate rigidity. To answer this question, first we checked the two hallmarks of cellular contractility *i.e.* actin stress fibre assembly and the formation of matured focal adhesions. As can be seen in Fig. 3A and the inset, hMSCs on a rigid substrate (40 kPa gel) produced prominent stress fibres (green) and large focal adhesions (red). When cultured on a soft substrate, no such thick stress fibres or large focal adhesions were formed as shown in Fig. 3B and the inset. However, when hMSCs were seeded at a high density on a soft substrate, the phenotypes were rescued and the formation of stable stress fibres and mature focal adhesions were observed (Fig. 3C and the inset). The presence of large focal adhesions also suggests that the cells were strongly adhered to the substrate and cell spreading (as shown in Fig. 1) was not merely based on cell–cell adhesion. The formation of mature focal adhesions and stress fibres indicates that at high seeding density on a soft substrate, cells exert high traction force, similar to cells on a stiff substrate.

To confirm this claim further, using traction force microscopy (TFM) we measured the deformation of a 500 Pa gel caused by the traction applied by an isolated cell and a pair of cells and the same for a cluster of cells by time-lapse imaging. Fig. 3D and E show the typical deformation heatmap of the gel surface caused by one and two cells respectively as obtained from TFM. Here, the white line shows the cell boundary. Fig. 3F shows the deformation caused by a cluster of cells when treated with LatB. We observed that upon treatment of LatB, the Hoechst stained nuclei moved from their original position (shown in red) to their final position (shown in grey) indicating relaxation of the underlying gel due to the loss of cellular traction (ESI Video S1†). From these three images, it was evident that the range of influence and the maximum value of substrate deformation both increased with increasing cell number as presented in Fig. 3G and H respectively. We found that the range of deformation by a single cell is about 80 μm (Fig. S1B†) which matches well with the observations made by earlier researchers.^13^ Next we processed the deformation data to obtain the traction stress as represented in Fig. 3I. A total of six such single cells or cell pairs were examined and the average traction force was plotted. Our analysis shows that cell pairs apply significantly higher traction force on a soft substrate than single cells. While it is computationally challenging to measure the traction force for multiple cells or cell clusters, we predict that the traction would be higher commensurating with higher deformation of the substrate (Fig. 3H).

### Cell crowding rescues cell cycle arrest induced by a soft substrate

**Fig. 4:**
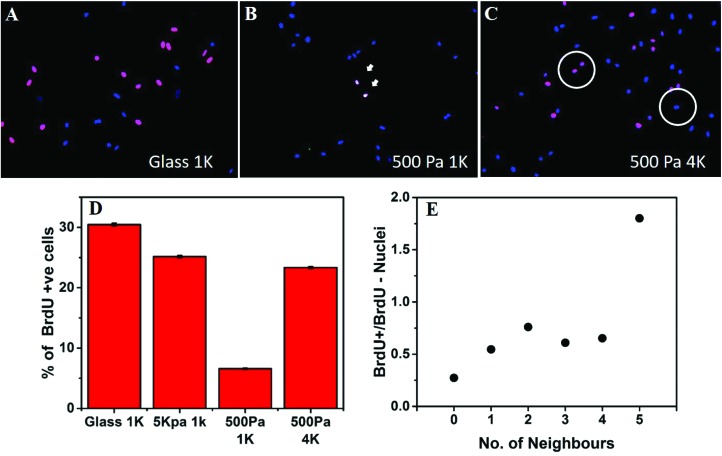
Increasing seeding density reverses the effect of substrate stiffness on cell cycle progression. (A–D) BrdU incorporation assay shows that a soft substrate arrests cell cycle progression. However, at a high seeding density, BrdU uptake is high even on a soft substrate indicating a rescue from cell cycle arrest. (DAPI: blue and BrdU+: magenta) (Mean ± standard error, *n* ≥ 900) (E) indicates that cells with more neighbours have a higher chance of overcoming the effect of substrate and progressing through the cell cycle (*n* = 400).

Next we asked the question whether the inter-cellular distance influences only the cellular morphology or it modifies cellular functions as well. For this purpose, we checked the combined effect of substrate stiffness and the intercellular distance on cell cycle progression. We used the BrdU incorporation assay to monitor DNA synthesis (Fig. 4A–C). When hMSCs were cultured on rigid substrates such as glass coverslips or 5 kPa gels, at a low seeding density (1000 cells per cm^2^), they showed a high level of DNA synthesis (Fig. 4A and D). However, when cultured on a soft gel (500 Pa) for 48 hours at the same density, the percentage of S phase cells dropped significantly (Fig. 4B and D). Interestingly, when the seeding density was increased to 4000 cells per cm^2^, cells no longer remained arrested despite being on a soft gel (Fig. 4C and D). To rule out the possible role of increased paracrine concentration, we diluted the media for the wells containing a higher number of cells. However, that did not alter our observation (data not shown).

We counted the number of neighbours that a given nucleus had within the two-cell diameter distance (60 μm, circle in Fig. 4C) and grouped nuclei according to the number of such near neighbours observed. For each group, the ratio of BrdU+ to BrdU– nuclei was calculated and plotted against the number of neighbours. We found that the ratio of cells progressing through the S phase increased with the number of neighbours, signifying that cell cycle progression on a soft substrate depends on the distance from the neighbouring cells. Data presented here come from a single experiment. However, repeated experiments showed the similar trend, as can be seen in Fig. S7.†

### Cellular traction causes local stiffening of the substrate

**Fig. 5:**
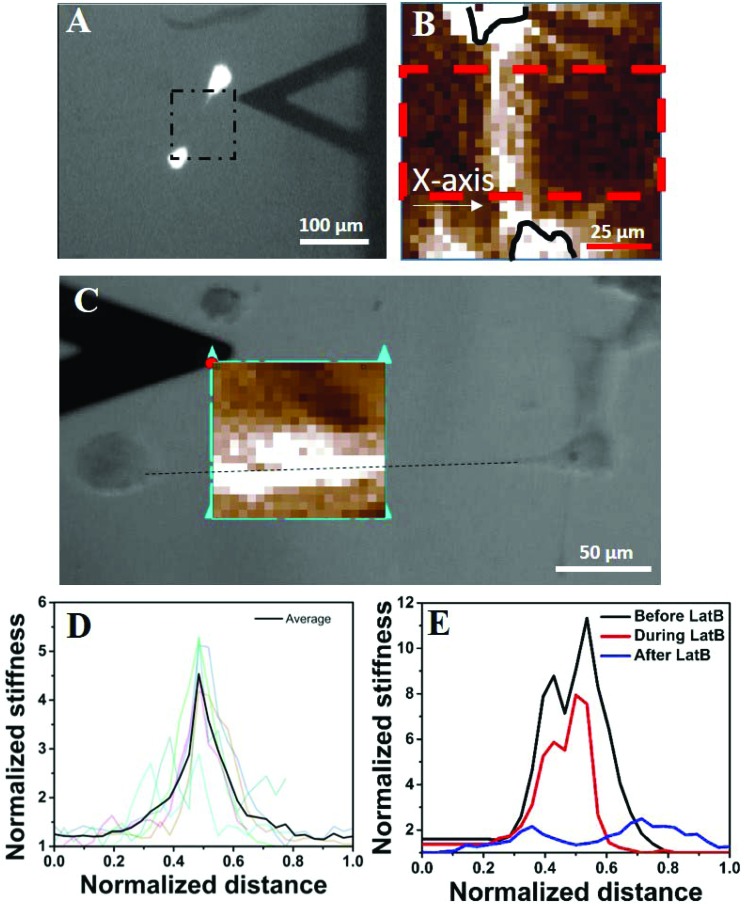
Cellular traction increases the local stiffness of the substrate. (A) AFM to measure the local stiffness of the gel between two cells. The cells are stained with calcein AM and hence appear bright. The square with a black dashed line represents the area scanned by the AFM tip. One of such representative scanned areas is shown in (B). (B) Represents a typical AFM force map showing two cell bodies (outlined in black solid lines) and the intermediate space. Light colours represent higher substrate stiffness. Stiffness values were averaged over the area marked by a red dashed line and represented in Fig. 3D. (C) The increase in apparent stiffness happens along the straight line joining the protrusions of two neighbouring yet non-touching cells. (D) AFM data for six different cell pairs are shown with light colour lines and their average value is shown using a dark line. The length is normalized with the length of the AFM scan along the *X*-axis as shown in Fig. 3B and the substrate rigidity is normalized with the basal value of the corresponding substrate as measured by AFM. (E) Shows that upon application of LatB, the increased rigidity (black line) of the PAA gel reduces in a time dependent manner (red line) and finally comes down at the basal value (blue line).

Next, we wanted to understand why an isolated cell responds differently towards the substrate rigidity (soft substrate in particular) compared to when it has one or more neighbours. Our observations that the global concentration of cytokines does not play a role (Fig. S3†) and the cells protrude preferentially towards their nearest neighbour in a distance dependent manner (Fig. 2) made us believe that the local substrate mechanics plays a dominant role here. Although the PAA gel has been shown to be linearly elastic from the bulk measurements,^17^ we wanted to check whether it stiffens locally in response to cellular traction. We measured the modulus of the substrate between the two cells using atomic force microscopy (AFM) (Fig. 5A). The cells were stained with cytoplasmic dye calcein AM for better visualization. The black triangle, seen in the images of 5A and 5C, is the AFM tip. The calcein AM stained bright cell bodies show that the cells were far apart and did not have any observable physical connection. We had checked this claim with higher magnifications as well. The square enclosed by the dashed line shows the approximate area on which AFM measurements were taken. The resulting modulus heat map is presented in Fig. 5B in which cell bodies are outlined with black continuous lines and the brighter colours represent the increasing modulus of the gel. The image clearly demonstrates a local increase of stiffness of the substrate along the line joining the protrusions of two cells. When the cells had protrusions (DP+), this apparent increase in stiffness took place along the straight line joining the protrusions, as shown in Fig. 5C. To note, here the neighbouring cells were more than 250 μm apart (cell center to cell center) eliminating the possibility of a direct contact. To quantify the apparent increase in stiffness, we took AFM measurements for six different cell pairs, the result of which is presented in Fig. 5D as normalized average. We indeed observed an increase of local stiffness by 5 times that of the unstrained gel. However, this fold change became reduced as the substrate stiffness increased (Fig. S8†). To confirm the hypothesis that this apparent increase in stiffness was indeed due to cellular traction, we further treated the cells with latrunculin B (LatB), a pharmacological inhibitor of actin polymerization, to reduce the traction force. We observed that the increased modulus indeed dropped to its base value in a time dependent manner as LatB gradually acted upon the actin stress fibre assembly (Fig. 5E).

This particular observation proves that the substrate stiffness indeed increases in response to cellular traction. However, one may argue that the increased stiffness observed here is a result of ECM deposition. We do not think so because of three reasons. First, the increased apparent stiffness can be observed within 45 minutes of cell seeding (Fig. S9†). This duration is too small for any considerable ECM deposition to take place. Second, if ECM deposition was the cause of increased gel stiffness then that should have happened as a radial outward gradient from the cell's body and not as a straight line or a rectangular strip as our AFM measurement shows (Fig. 5B and C (bright colours)). Third, if increased gel rigidity was due to ECM deposition then that would not have reduced to its basal level within 20 minutes of treatment with the actin inhibitor LatB (Fig. 5E).

Another possible explanation for the increased stiffness of the region joining two cells can be the formation of an actin structure, such as a nanotube or cytoneme. However, we could not observe any such structure even after a careful search under an optical microscope. The possibility of any structure thinner than the limit of the optical microscope can also be ruled out as the width of the increased stiffness is ∼25 μm, as can be observed in Fig. 5C.

To summarize the results, here we described that many well-documented effects of the soft substrate on cellular behaviour become reversed when cells are plated at a high seeding density. Such cellular behaviour includes morphology, focal adhesion maturation, stress fibre formation, cellular traction and proliferation. We believe that these effects arise from the strain created by the neighbouring cells.

It is not unknown that adherent cells can sense strain in the substrate caused either by a neighbouring cell or by any other external means such as substrate stretching.^24^ For example, Lo *et al.* showed that fibroblasts on a soft PAA gel can sense substrate deformation caused by a micro-pipette and move towards it if the strain is tensile and away from it if the strain is compressive.^22^ Similarly, multiple authors have shown that cells reorganize themselves on a soft substrate if an external strain is applied.^24^ Researchers have also demonstrated both computationally and experimentally that when cells are seeded at a high density on a soft substrate they align and form strings and networks. Recently, it has been shown that keratinocytes when seeded at a high density on a soft substrate form colonies.^25^ However, the underlying mechanism of such inter-cellular communication *via* a soft matrix is still debated.

For non-linear strain stiffening gels such as collagen or fibrin, an increase in apparent stiffness due to cellular strain has been implicated for such density dependent behaviour. In many computational studies, the neo-Hookean model was used to capture non-linearity in the material properties of the substrate.^12^ However, bulk measurements of rheological properties have shown the PAA gel to be linearly elastic for a large strain limit. Still the question remains: can the PAA gel though globally linearly elastic be locally non-linear in nature, as shown by Boudou *et al.* using the micropipette aspiration technique.^26^ They have shown though the PAA gel is known as linearly elastic, may not behave so when locally strained. Other recent studies have shown that the PAA gel may have significant viscous behaviour as well.^27^ Our AFM data support this view as we observed a local increase in stiffness which was controlled by cellular traction. Such local stiffening can very well explain why cells extend preferentially towards their neighbours finally forming a network pattern. When seeded on a soft substrate, a cell randomly extends its filopodia in all directions and probes the stiffness of its surroundings. As soon as a region that can resist the contractile pull is found, the cell reinforces the focal adhesion at that site, forms stress-fibres, increases contractility in that direction, and finally spread preferentially towards this rigid region.^28^ However, if two cells are close enough, during the random search for stiff regions, they may land up pulling on the same region of the substrate causing a local stiffening of the soft gel as demonstrated, which in turn may result into preferential spreading. The observation that the network formation starts when the average distance between the cells is less than 150 μm (Fig. S1†) indirectly supports this hypothesis as the range of substrate deformation by a single cell is ∼75 μm, half of the average distance (Fig. S1†).^13^

Two observations of this paper demand further discussion. The first one is the generation of a cellular pattern due to the cooperative behaviour of hMSCs over a soft substrate. Maintaining long-range synchronization and establishing an order is crucial in pattern formation during embryogenesis.^29^ While the importance of morphogen gradients has long been appreciated in tissue patterning, the role of mechano-signals has been started to get investigated recently.^30^,^31^ The observation that the tension created by cellular contractility causes a global patterning in the developing embryo^32^ brings direct cell–cell and matrix-mediated interactions to the center stage in investigating the pattern formation and morphogenesis. In our study, we observed that depending on the density, cells form various patterns on soft substrates (Fig. S4†). According to the model proposed by Bischofs and Schwartz, when seeded on a soft substrate, the strain field created by one cell is sensed by its neighbour, which responds by reorienting itself and spreading along the major axis of the strain field.^33^ Re-orientation of MSCs in response to anisotropic cyclic stretching of the substrate has also been demonstrated experimentally in the context of cellular differentiation.^33^,^34^ In the present study, although there is no externally applied strain, two neighbouring cells align their principal axis to synchronize their self-generated strain field. When seeded on a soft substrate, a cell randomly extends its filopodia in all directions and gauges the stiffness of its surroundings. As soon as a region that can resist the contractile pull is found, the cell reinforces the focal adhesion at that site, forms stress-fibres, increases contractility in that direction, and finally spreads preferentially towards this rigid region.^28^ However, if two cells are close enough, during the random search for stiff regions, they may land up pulling on the same region of the substrate causing either a real local stiffening of the gel as suggested here or a perceived stiffening due to opposing deformation caused by the neighbour. This phenomenon may result into preferential spreading. Also, our results point out that such pattern formation is not limited to endothelial cells as observed by earlier researchers but are more universal.^14^,^15^ As we have shown in supplementary Fig. S2,† 3T3 fibroblast and C2C12 myoblast too showed a similar phenotype supporting our hypothesis indirectly that the observed phenomenon is mainly governed by the substrate mechanics and not by cell type specific biology.

The other observation that needs further attention is increased proliferation. For the first time, we demonstrate the combined effect of the substrate and inter-cellular distance on any cellular function, here proliferation. Earlier studies demonstrated that contractility is essential for cell proliferation.^35^ At a lower seeding density, cells cannot exert contractile force on the soft substrate and thus cannot proliferate. However, when plated above a threshold density, we have shown that cells can mechanically interact with each other *via* the substrate and that causes them to exert contractility and start proliferating. As more cells divide, the cell number and the overall strain on the gel surface increase converting the soft and deformable gel surface into a taut, stretched elastic membrane. As a result, the effective mechanical micro-environment changes from soft to stiff and thus causes changes in cell morphology and behaviour. This observation indicates that a local increase in the cell number (or decrease in cell–cell distance) may switch the cell fate from quiescence to proliferation which may have significance in the loss of homeostasis in our body during many diseases.

Earlier studies have shown that hMSC differentiation depends both on substrate stiffness as well as on the seeding density. hMSCs differentiate into the adipogenic lineage when cultured either at a low seeding density on soft substrates or at a high seeding density on stiff substrates. On the other hand, osteogenesis is preferred when hMSCs are cultured on stiff substrates at a low seeding density.^36^,^37^ Our results suggest that these observations may get modified if cells are cultured at a high seeding density on a soft gel. Does the effect of seeding density on cellular differentiation also depend on substrate stiffness? Many such questions related to hMSCs’ response towards substrate rigidity may deserve a re-visit to understand the interplay of substrate mechanics and cell density.

## 5. Summary and conclusion

In this paper, we have shown that hMSCs (i) spread more, (ii) form mature focal adhesion and stress fibres, (iii) applied higher traction and thus deform the substrate more and (iv) show higher proliferation when cultured on a soft PA gel at a high density than when cultured sparsely. Our AFM data, with and without the pharmacological inhibitor LatB, indicate that at a high seeding density, hMSCs probably stiffen the gel locally due to the applied strain. This local stiffening might be the cause of the cooperative behaviour of hMSCs on the soft gel as observed by us and others.^26^

To conclude, this paper shows that *in vitro* studies of cell–substrate interactions are critically dependent on inter-cellular distances. Typically in tissues, the cell density is much higher than that is used for *in vitro* studies of mechano-signaling. It is therefore important to explore how cell–cell mechanical interaction *via* the ECM matrix can modulate the properties of the matrix itself, causing a feed-back change in cell behavior, both in the physiological matrix as well as synthetic materials.

## Conflicts of interest

There are no conflicts to declare.

## Supplementary Material

Supplementary information
