# Supplementary material for: Cell density overrides the effect of substrate stiffness on human mesenchymal stem cells’ morphology and proliferation
Source: Biomater Sci. 2018 Mar 12;6(5):1109–19. doi: 10.1039/c7bm00853h (PMC5933002; doi:10.1039/c7bm00853h)
Supplement: Supplementary file 1 [file BM-006-C7BM00853H-s001.pdf]

Electronic Supplementary Material (ESI) for Biomaterials Science.
This journal is © The Royal Society of Chemistry 2018

Supplementary Materials

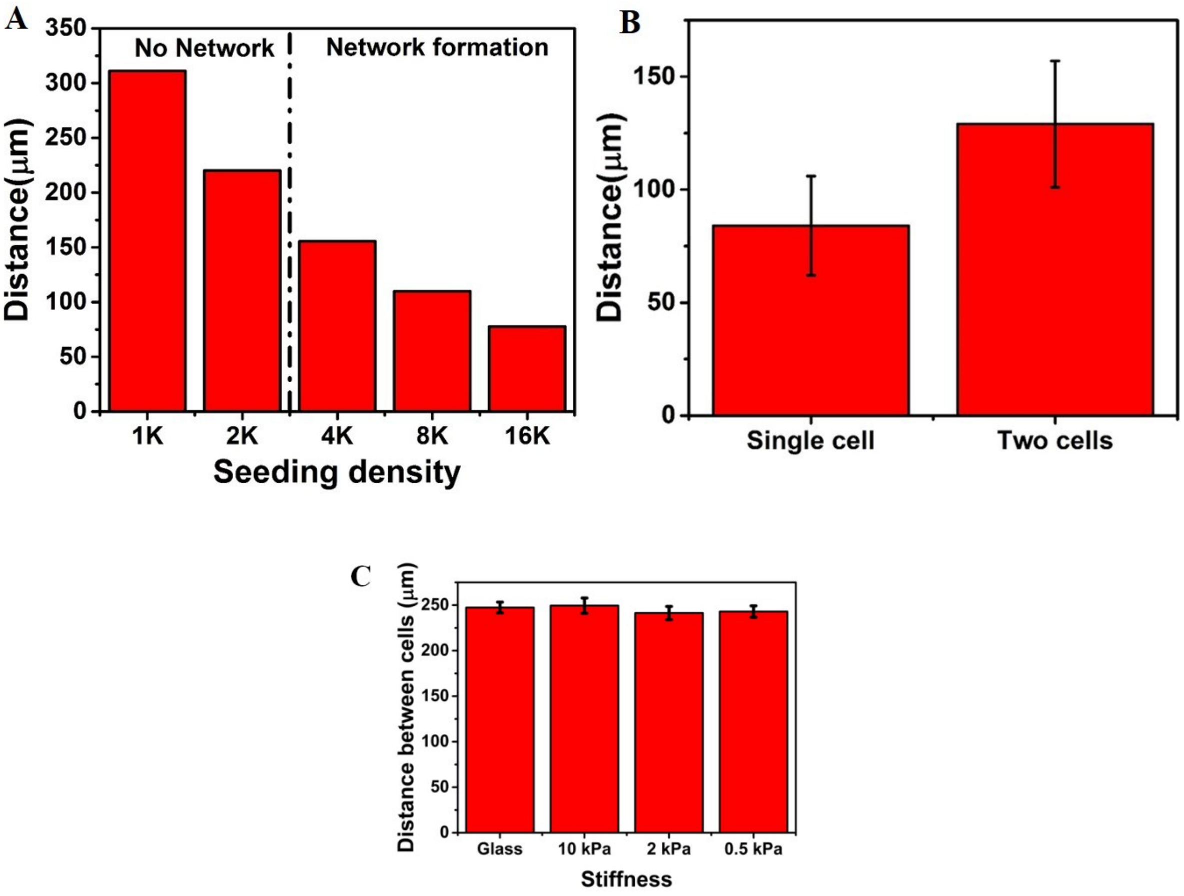

Figure S1: Distance between the cells decreases as the seeding density increases: *(A)The bar graph shows the calculated average distance between the two cells as the seeding density of the cell is increased from 1000 cells/cm² to 16,000 cells/cm². (B) The radius of the gel deformation region for single and two cells (n=6). (C) hMSCs were seeded at 2000 cells/cm² on the substrates of stiffness of 0.5, 2, 10 kPa, and glass. The average distance between two cells were estimated from images taken after 4 hours of cell seeding. It shows that number of MSCs that adhere to substrate was not stiffness dependant (N=3, n>90 images).*

With increasing seeding density, the average distance between two cells decreases, as shown in graph of figure S1A. At low seeding density, such as 1K/cm² the average distance between two cells is approximately 300 μm which reduces to 150 μm or less at seeding density of 4K or higher. In our experiments, we could observe the formation of cellular network only at 4K seeding density or beyond. Lower than that seeding density, no global pattern formation was observed. The graph of

figure S1B shows the range of substrate deformation (at least 2μm of bead displacement obtained from TFM) of a single cell is around 75 μm which is in accordance with.[1] As the cell number increased from one to two, this range increased to 125 μm meaning deformation caused by two closely placed neighbours will reach the third cell which is within that range. As shown in the figure S1A, for seeding density more than 4K, average distance between two cells falls within this range leading to initiation of network formation.

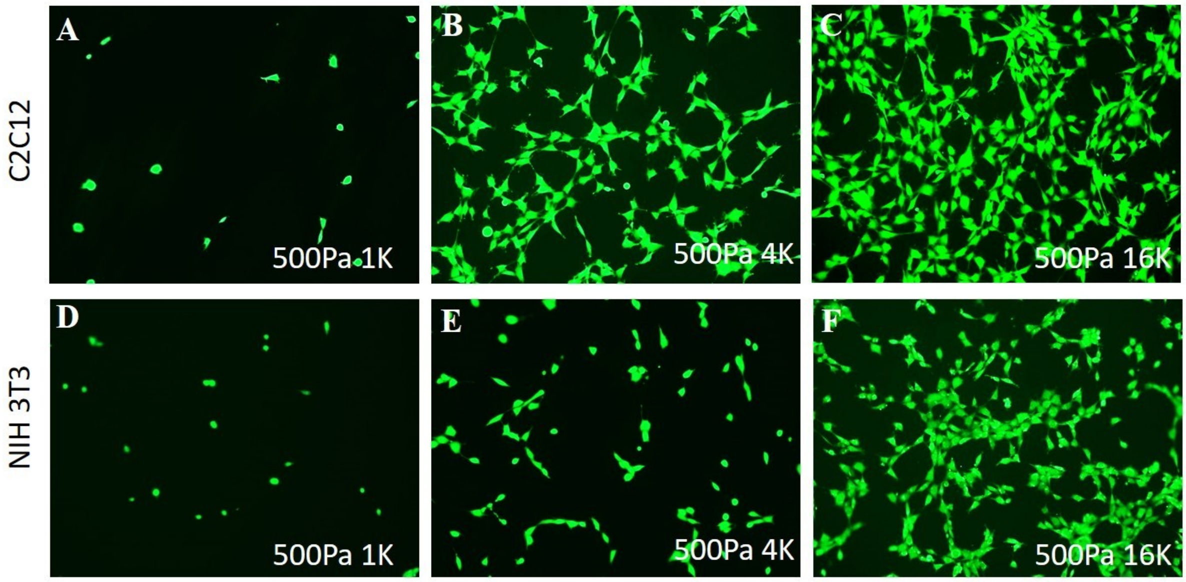

Figure S2: Seeding density effect is independent of cell type: *Fluorescent images (A-C) C2C12 cells and (D-F) NIH 3T3 cells on stiffness of 500Pa with seeding density of 1K(A), 4K(B) and 16K (C).*

Although we were able to observe the formation of the cellular networks with hMSCs, and literature shows the network formation with endothelial cells,[2] we wanted to check if the observation is cell type specific. To address this question, NIH 3T3 and C2C12 were cultured on the different substrates (500Pa, 1 kPa, 2 kPa, and glass) with different seeding densities. It was observed that both cell types formed the networks from seeding 4K seeding density onwards as shown in the figure S2.

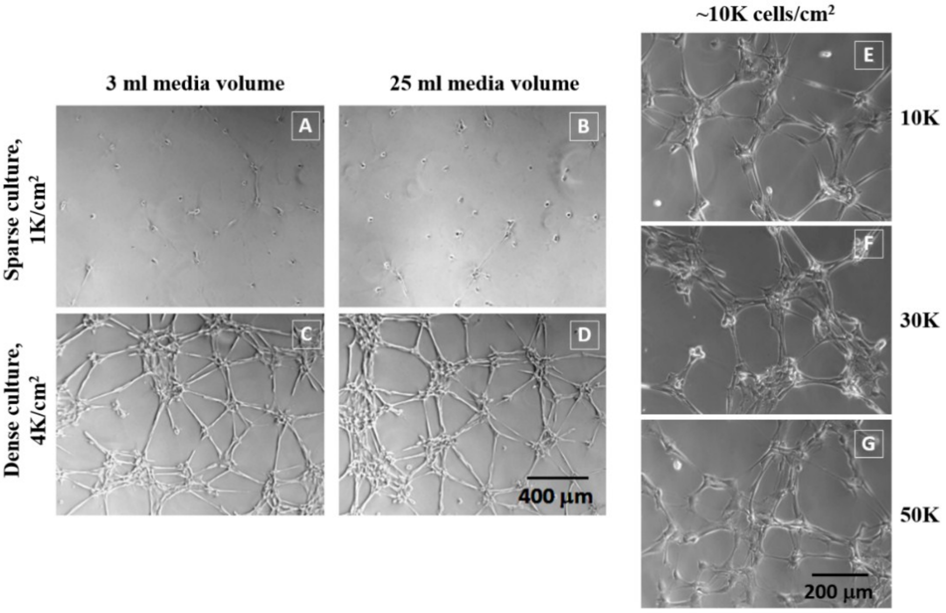

Figure S3. Network formation is independent of global growth factor/cytokine concentrations. *Cells were cultured at two different seeding densities 1K cells/cm² (A-B) and 4K cells/cm² (C-D) with media volume of 3 ml (A and C) and 25 ml (B and D). From A to D, although cell density was increased by 4 times, the media volume was increased by more than 8 times, making an increase in the global concentration of cytokines unlikely. In figure (E-G), total cell number was increased from 10K to 50K keeping seeding density constant by changing seeding area. We observe that formation of networks is independent of total number of cells thus total cytokine concentration.*

With increased seeding density, not only does intercellular separation distance decreased, but the concentration of secreted molecules increased, which might had an effect on individual cell spreading and the formation of global patterns. To test that possibility, we performed two experiments. First, we increased the media volume by 8 times while keeping cell density constant expecting that any excess release of growth factors/cytokines would be diluted (fig S3A-D). No change in network formation was observed for dense cultures (fig S3C and 3D) which confirmed our assumption that cell density or intercellular distance is the more important parameter, rather than increased cytokine release by the greater number of cells. To further confirm this observation, the seeding density was kept constant at 10K/cm² but total cell number was decreased from 50K to 10K by proportionately reducing total seeding area (fig S3E-G). Again, there was no appreciable change in network formation. However, 10K cells seeded on 5 cm² gel at a density of 2K/cm² fail to spread and form network.

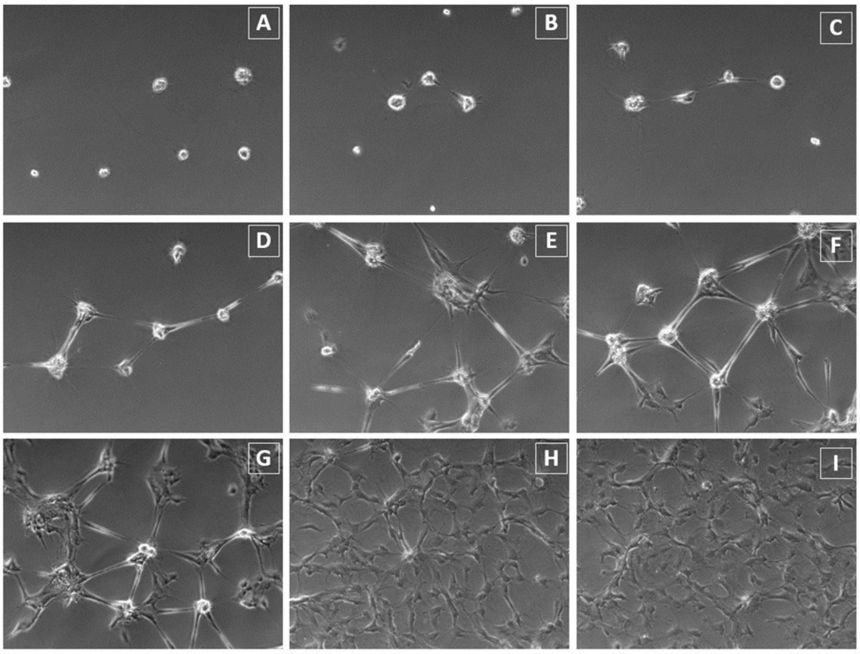

Figure S4: Formation of different global patterns depending on seeding density. *As cell density increases, isolated cells with round morphology (A), begin formation of directed protrusions (B), strings (C), open (D and E) and eventually create closed networks (E and F) and monolayers (H and I).*

With the increase in the seeding density, the average distance between the two cells decreases. At sparse seeding density, the cells tend to remain round as shown in the image S4 A. As the distance between the two cells decreases (approximately 100-200 µm) they start to form the protrusion towards the neighbouring cell as shown in figure S4 B. The formation of the protrusions can be carried out by the other neighbouring cells leading to the formation of the chain of the cells similar to the beads and string as shown in the image S4 C. As the seeding density is increased the formation of the directed protrusions leads to the formation of the highly complex patterns as shown in the figure E, F, and G. However, when the cell number is increased furthermore the cells start to form a complete sheet of cells and cover the whole surface by forming a monolayer as shown in images S4 H-I.

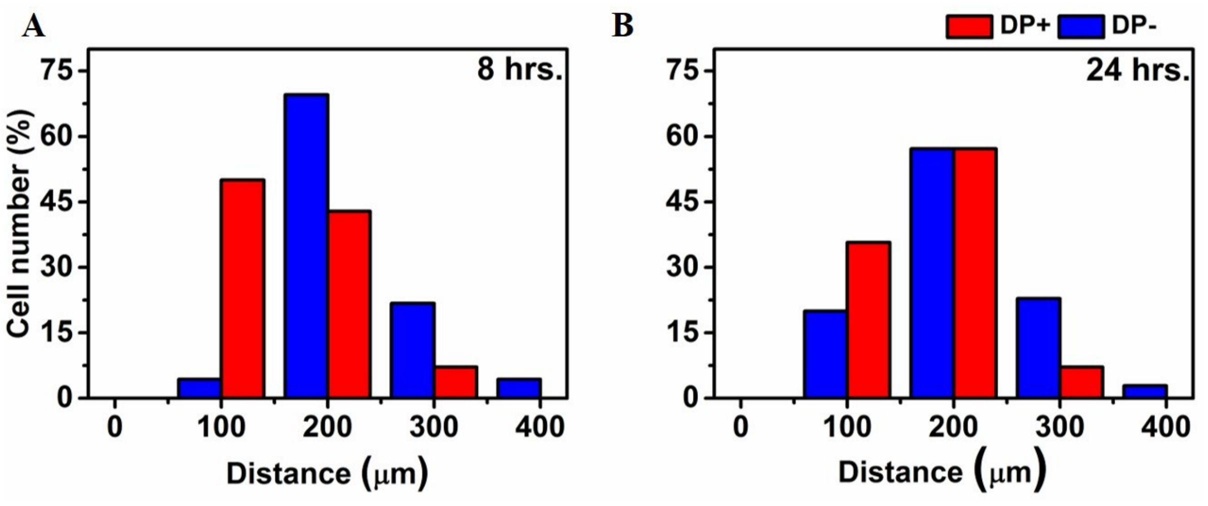

Figure S5: Protrusions shows biasness towards the neighbouring cell. *The bar graph shows the percentage distribution of DP+ and DP- cells with intercellular distance. (A) distribution after 8 hrs of seeding, (B) distribution after 24 hrs of seeding.*

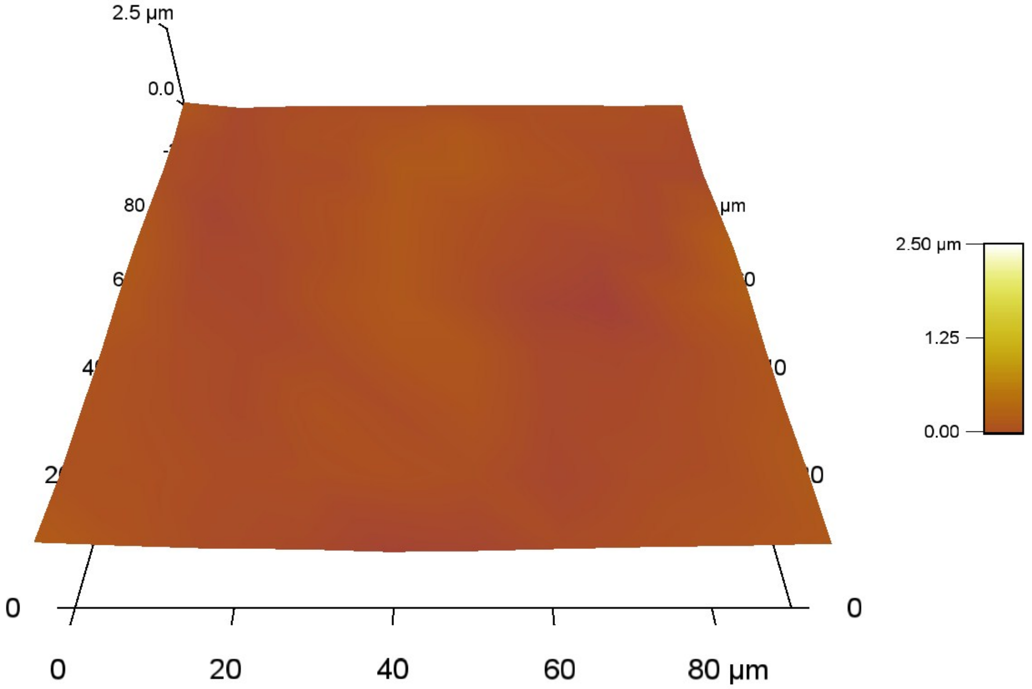

Figure S6: Topology of the gel surface as shown by AFM. *The image shows the topology of the gel (500 Pa) for 90 µm * 90 µm scan area obtained from AFM.*

After removing of the polymerized gel from the hydrophobic surface, the top surface of the gel being used for the cell culture remained undulated. The figure S6 shows the afm topology of the gel (500 Pa) for a region of 90 µm * 90 µm. It can be seen that the surface remained at the base level of 0 µm. This confirms that the network formed by the cells is not inflenced by the topology of the gel surface.

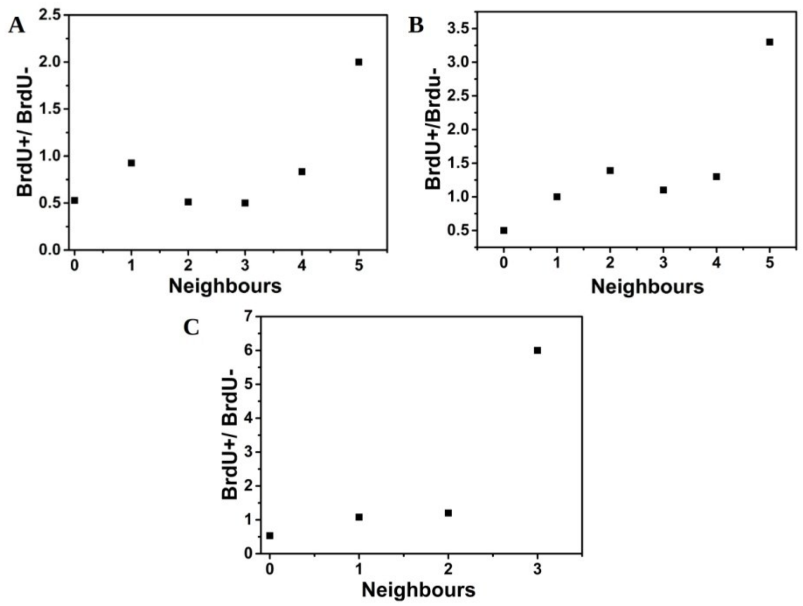

Figure S7: More number of neighbours helps to prevent soft substrate induced cell cycle arrest. *The scatter plot shows the ratio of BrdU+ cells to BrdU- cells with respect to the number of neighbours that a particular cell had in its vicinity. Graphs A), B), and C) , obtained from three independent experiments, show that ratio of BrdU+ cells over BrdU- cells increases with the number of neighbours, albeit non-lineraly.*

The cells were seeded onto the gel of 500 Pa with different experimental conditions. The cells were given the BrdU pulse and quantified for BrdU presence (BrdU+) or absence (BrdU-). The number of cells present in the diameter of 60 μm of BrdU+ or BrdU- cells were calculated and their ratio's were plotted as shown in figure S7. It can be seen that in all three of the independent experiments, the proportion of BrdU+ cells with respect to BrdU- cells increased with increasing number of neighbour cells.

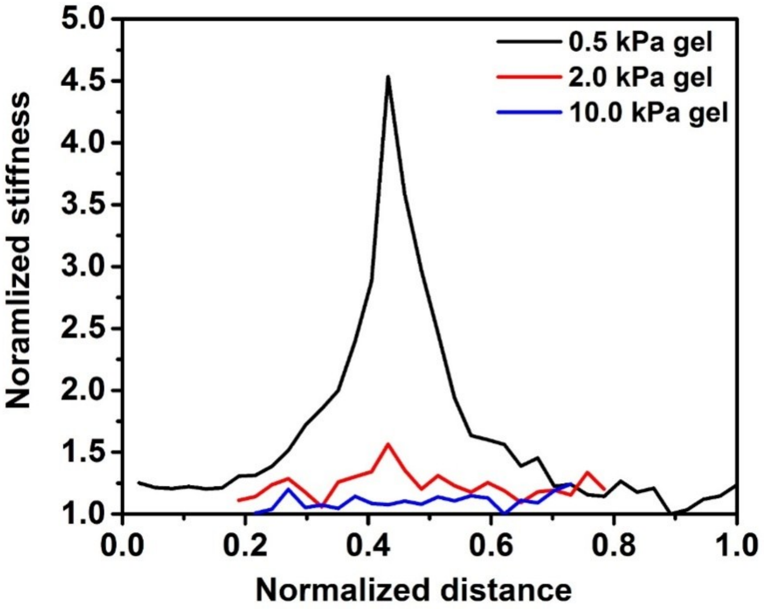

Figure S8: Decrease in stiffness fold change with increasing substrate stiffness. *The line graph shows the increase in the stiffness between two cells seeded onto different substrates 500Pa (black line), 2 kPa (red line), and 10 kPa (blue line).*

While soft gels (500 Pa) in response to deformation by neighbouring cells showed about 5 fold increase in apparent stiffness as shown by the black solid line (Figure S8), stiffer gels did not show such prominent change. 2 kPa gel showed a small peak with increase in apparent stiffness up to 1.5 times (red line). For gel of stiffness10 kPa, the stiffness between did not change at all (blue line).

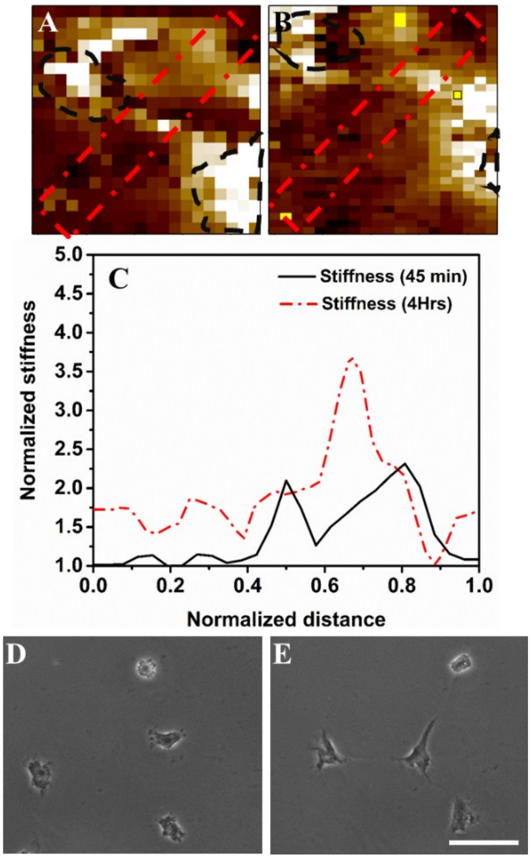

Figure S9: Intermediate stiffness between two cells increases even during the initial period of cell culture and helps in making protrusions. *The top row shows the heat map of 90 µm * 90 µm scan area of the gel (500Pa) with cells shown with black dotted lines. The heat maps show the same cell set (A) after 45 minutes of seeding and, (B) after 4 hours of seeding the cells. The red dotted rectangle shows the region used to calculate the average stiffness shown in image C. (C) Shows the increase in the stiffness of the substrate between two cells at 45 minutes and 4 hours of seeding. Image (D) and (E) shows the representative image of the morphology of cells at 50 minutes and 4 hours of seeding respectively. The scale bar is 100 µm.*

During the initial hours of seeding the cells interact with the surrounding, and forms protrusions towards its neighbour in the vicinity as explained earlier. AFM measurements done during the initial hours of seeding showed that within 45 minutes of seeding the cells starts to orient towards the neighboring cells. Due to this there is a small increase in the stiffness of the gel in between the two cells as measured for figure S9 A and shown by black solid line in figure S9 C. The stiffness showed an increase around two fold compared to the base stiffness at 45 minutes of seeding. As the time

lapsed, the stiffness between the two cells increased as measured for figure S9 B and shown by red dotted line of figure S9 C. After 4 hours of seeding the stiffness changed from 2 fold to around 3.7 times the base value. As mentioned earlier, the formation of network begins within 4 hours of seeding. Image S9 D and S9 E shows the phase image of the cells at 50 minutes and 4 hours after seeding. During the initial hours of seeding the cells remained round (figure S9 D) but as the time progressed they started to make protrusions towards the neighboring cell (figure S9 E).

References

1 C. A. Reinhart-King, M. Dembo and D. A. Hammer, *Biophys. J.*, 2008, **95**, 6044–6051.

2 J. P. Califano and C. A. Reinhart-King, *Cell. Mol. Bioeng.*, 2008, **1**, 122–132.
